# Using Upper Arm Vein as Temporary Pacemaker Access Site: A Next Step in Minimizing the Invasiveness of Transcatheter Aortic Valve Replacement

**DOI:** 10.3390/jcm13030651

**Published:** 2024-01-23

**Authors:** Maxim J. P. Rooijakkers, Geert A. A. Versteeg, Marleen H. van Wely, Laura Rodwell, Lokien X. van Nunen, Robert Jan van Geuns, Leen A. F. M. van Garsse, Guillaume S. C. Geuzebroek, Michel W. A. Verkroost, Robin H. Heijmen, Niels van Royen

**Affiliations:** 1 Department of Cardiology, Radboud University Medical Center, 6525 GA Nijmegen, The Netherlands; max.rooijakkers@radboudumc.nl (M.J.P.R.); geert.versteeg@radboudumc.nl (G.A.A.V.); marleen.vanwely@radboudumc.nl (M.H.v.W.); lokien.vannunen@radboudumc.nl (L.X.v.N.); robertjan.vangeuns@radboudumc.nl (R.J.v.G.); 2 Section Biostatistics, Department of Health Sciences, Radboud Institute for Health Sciences, 6525 EZ Nijmegen, The Netherlands; laura.rodwell@radboudumc.nl; 3 Department of Cardiothoracic Surgery, Radboud University Medical Center, 6525 GA Nijmegen, The Netherlands; leen.vangarsse@radboudumc.nl (L.A.F.M.v.G.); guillaume.geuzebroek@radboudumc.nl (G.S.C.G.); michel.verkroost@radboudumc.nl (M.W.A.V.); robin.heijmen@radboudumc.nl (R.H.H.)

**Keywords:** aortic stenosis, bleeding complications, pacemaker, transcatheter aortic valve replacement

## Abstract

**Background** The femoral vein is commonly used as a pacemaker access site during transcatheter aortic valve replacement (TAVR). Using an upper arm vein as an alternative access site potentially causes fewer bleeding complications and shorter time to mobilization. We aimed to assess the safety and efficacy of an upper arm vein as a temporary pacemaker access site during TAVR. **Methods** We evaluated all patients undergoing TAVR in our center between January 2020 and January 2023. Upper arm, femoral, and jugular vein pacemaker access was used in 255 (45.8%), 191 (34.3%), and 111 (19.9%) patients, respectively. Clinical outcomes were analyzed according to pacemaker access in the overall population and in a propensity-matched population involving 165 upper arm and 165 femoral vein patients. Primary endpoint was Bleeding Academic Research Consortium (BARC) type 2, 3, or 5 pacemaker access site-related bleeding. **Results** In the overall population, primary endpoint was lowest for upper arm, followed by femoral and jugular vein access (2.4% vs. 5.8% vs. 10.8%, *p* = 0.003). Time to mobilization was significantly longer (*p* < 0.001) in the jugular cohort compared with the other cohorts. In the propensity-matched cohort, primary endpoint showed a trend toward lower occurrence in the upper arm compared with the femoral cohort (2.4% vs. 6.1%, *p* = 0.10). Time to mobilization was significantly shorter (480 vs. 1140 min, *p* < 0.001) in the upper arm cohort, with a comparable skin-to-skin time (83 vs. 85 min, *p* = 0.75). Cross-over from upper arm pacemaker access was required in 17 patients (6.3% of attempted cases via an upper arm vein). **Conclusions** Using an upper arm vein as a temporary pacemaker access site is safe and feasible. Its use might be associated with fewer bleeding complications and shorter time to mobilization compared with the femoral vein.

## 1. Introduction

Aortic stenosis (AS) is the most common valvular heart disease in developed countries. Randomized trials have demonstrated the noninferiority or even superiority of transcatheter aortic valve replacement (TAVR) compared with surgical aortic valve replacement in patients with severe AS across the spectrum of surgical risk [1,2,3,4,5,6].

During TAVR procedures, a temporary pacing lead is often used for rapid ventricular pacing. This is indicated during pre- and post-dilation. Moreover, pacing provides a direct back-up in the case of procedural conduction disturbances. Post-TAVR, the temporary pacing lead is frequently left in place to overcome conduction disturbances for up to 24 h, depending on periprocedural electrocardiographic features [7].

In current practice, the femoral vein is the most commonly used temporary pacemaker access site in patients undergoing TAVR under local anesthesia with or without conscious sedation. In patients undergoing TAVR under general anesthesia, the jugular vein is often used as this is the preferred access site by anesthesiologists in some centers. Both the femoral and jugular vein are prone to access site hematomas due to the anatomic position and large vessel diameter, occurring in approximately 4–10% of patients [8,9]. In addition, patients with femoral- or jugular-inserted temporary pacing leads are unable to mobilize while the pacing lead is still in place to prevent dislocation of the lead. Prolonged immobilization is a well-established risk factor for postoperative delirium (POD) and urinary tract infections [10,11], which in turn may lead to prolonged hospitalization with inherently increased healthcare costs.

The use of an upper arm vein as a temporary pacemaker access site is emerging, but it has not yet been evaluated in a prospective manner. In this study, we assessed the safety and efficacy of an upper arm vein as a temporary pacemaker access site during TAVR. We hypothesize that upper arm vein access might lead to fewer access site hematomas and a shorter time to mobilization after TAVR compared with either femoral or jugular vein access.

## 2. Materials and Methods

### 2.1. Study Population

In this prospective cohort study, we examined all consecutive patients who underwent TAVR for severe symptomatic AS at the Radboud University Medical Center between January 2020 and January 2023. Furthermore, we performed a propensity score (PS)-matched analysis of only patients who underwent transfemoral TAVR with the use of an upper arm or femoral vein as a temporary pacemaker access site. Any patients who required a cross-over of the pacemaker access site were excluded from the PS matching procedure. Treatment allocation according to current guidelines on the treatment of AS was performed by a dedicated heart team. The local Medical Research Ethics Committee provided a waiver since this study did not require an ethical review.

### 2.2. Procedure

All TAVR procedures were performed according to routine local protocol. Procedures were performed in a hybrid catheterization laboratory with an operating team consisting of an interventional cardiologist, a cardiothoracic surgeon, and a cardiothoracic anesthesiologist.

The use of an upper arm vein as a pacemaker access site was introduced in our center on 17 July 2020. Hereafter, data on pacemaker access sites were prospectively collected and stored in an electronic database (Castor EDC, Amsterdam, the Netherlands). In most cases of an upper arm approach for the temporary pacing lead, the superficial basilic vein was selected. However, due to anatomical variations, sometimes the brachial or cephalic vein was used to obtain venous access. The selection of which upper arm vein to puncture was left to the discretion of the operator, who normally chose the most robust vein (as identified with ultrasound). Over time, an upper arm vein access site was increasingly used and became the predominant pacemaker access site (Figure S1). All TAVR operators were skilled in placing the pacing lead through either an upper arm, femoral, or jugular vein. Importantly, the decision of which pacemaker access site to use was always left to the discretion of the operator. All patients that underwent transapical TAVR received a jugular vein pacemaker. Most patients that underwent transaxillary TAVR received a jugular vein pacemaker as well; however, due to operator preference and patient-specific characteristics, upper arm or femoral access could also be used.

**Figure 1:**
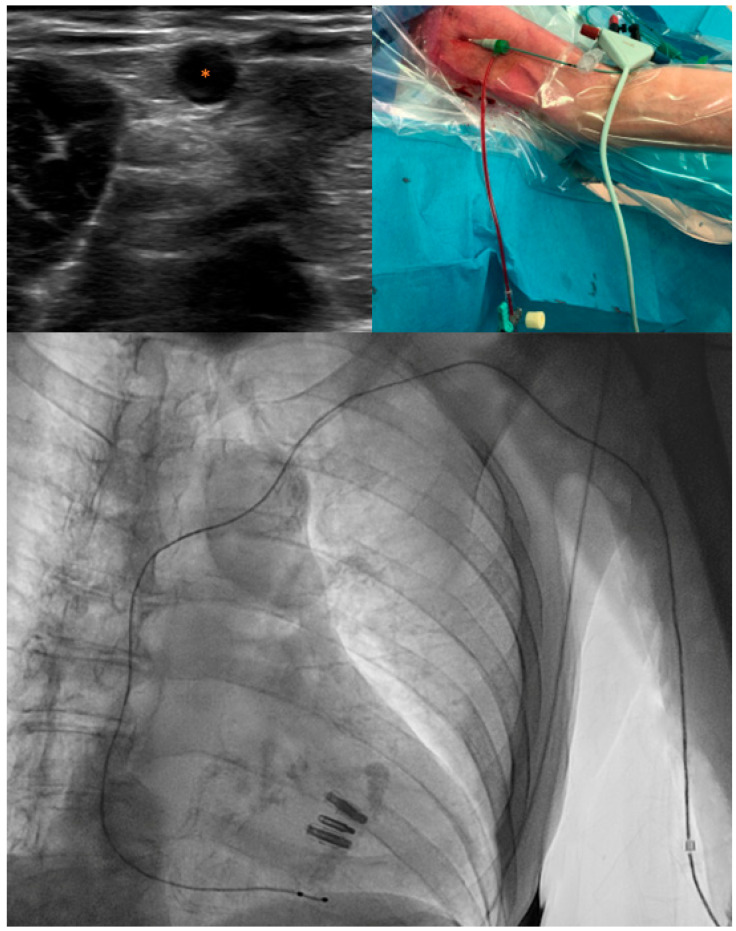
Example of a temporary pacing lead in a left upper arm vein. **Upper left**: Ultrasound-guided visualization of the vein for temporary pacemaker access. Asterisk denotes the vein. **Upper right**: 5F temporary pacing lead inserted through a 6F sheath in a left upper arm vein. **Lower**: Angiographic visualization of the temporary pacing lead trajectory from a left upper arm vein to right ventricular apex.

In patients in whom an upper arm vein was used as the pacemaker access site, a floating 5F pacemaker electrode (Pacel Bipolar Pacing Catheter; Abbott) was inserted through a 6F sheath in an upper arm vein (left or right, at the operator’s discretion) (Figure 1). In brief, the upper arm was placed in a downward hanging position at a 90-degree angle to the body. A tourniquet was placed around the upper arm (away from the disinfected area) to enhance the visualization of the upper arm veins with ultrasound. The veins running through the middle and distal parts of the upper arm were scanned to identify the most suitable vein for puncture. Robust veins without relevant anatomical structures (e.g., artery and nerves) in the proximity were considered most suitable for puncture. After the application of local anesthesia to the area around the selected upper arm vein, the vein was directly punctured with the needle from the introducer sheath kit (using ultrasound guidance). In most cases, this was the superficially running basilic vein, which was punctured slightly proximal to the elbow crease. In case the basilic vein was not easily accessible, the brachial or cephalic vein could be used as an alternative. After successful puncture of the vein, the guidewire was inserted through the needle, the needle was subsequently withdrawn, and the 6F sheath was advanced over the guidewire into the vessel. After insertion of the pacing lead through the sheath and correct placement of the lead in the right ventricle, the pacing lead and the sheath were attached to the upper arm using sterile fixation material (e.g., Tegaderm). In patients in whom the femoral vein was used as the pacemaker access site, the floating 5F pacemaker electrode was inserted through a 6F or 7F sheath in the femoral vein (left or right, at the operator’s discretion). Ultrasound guidance was used in all patients to assist in venous sheath placement, and angiographic guidance was used for placement of the temporary pacing lead in the right ventricle. Diagnostic access was obtained via a 6F sheath in either the femoral or radial artery at the operator’s discretion.

All patients underwent transfemoral TAVR with a self-expanding device (Medtronic Evolut R, Abbott Portico or Abbott Navitor) or a balloon-expandable device (Edwards Sapien III or Meril Myval). Pre- and post-dilation were left to the operator’s discretion. All pacemaker access site-related bleeding was assessed before leaving the catheterization laboratory, directly on arrival at the Cardiac Care Unit, and 24 h after TAVR. Clinical endpoints were prospectively collected according to the Valve Academic Research Consortium-3 (VARC-3) criteria [12].

### 2.3. Endpoints

The primary endpoint was clinically relevant pacemaker access site-related bleeding (bleeding academic research consortium (BARC) type 2, 3, or 5). Secondary efficacy endpoints included time to mobilization after TAVR, duration of hospitalization, skin-to-skin time, fluoroscopy time, temporary pacemaker dysfunction, need for back-up pacing, and cross-over of the pacemaker access site. Secondary safety endpoints included BARC type 2, 3, or 5 bleeding not related to the pacemaker access site and early safety (at 30 days) according to VARC-3 criteria. All endpoints were prospectively collected and stored using Castor.

### 2.4. Statistical Analysis

Continuous variables with normal distribution are presented as mean ± standard deviation and were compared using Student’s *t*-test (in the case of 2 groups) or an analysis of variance (ANOVA) model (in the case of 3 groups), followed by the Bonferroni test when findings with the ANOVA model were significant. Non-normally distributed continuous variables are presented as median with interquartile range (IQR) and were compared using the Mann–Whitney U test (in the case of 2 groups) or the Kruskal–Wallis test (in the case of 3 groups). Categorical variables are presented as a number (percentage) and were compared using the Chi-Square test.

To account for the non-randomized assignment to the pacemaker access site, two approaches were used. First, for the overall study population, baseline characteristics were tested in a univariate logistic regression model as predictors for the primary outcome (pacemaker access site-related bleeding). Variables with *p* < 0.2 were selected for the multivariate model. Selected variables were entered at the same time into a model, and the least significant variable was removed until only variables with a *p* < 0.05 were left.

Second, patients with a primary transfemoral access site for the TAVR and either an upper arm or femoral vein approach were separately analyzed. In this subset, PS matching was also performed. For 1:1 PS matching, optimal pair matching was performed using the MatchIt package in R [13], which calls functions from the optmatch package [14]. The matching is optimal in the sense that the sum of the absolute pairwise distances in the matched sample is as small as possible. The balance between groups was considered adequate if the absolute value of the mean standardized difference did not exceed 10%. Matching was performed on the following baseline variables associated with bleeding after TAVR [15,16,17,18]: age, sex, European System for Cardiac Operative Risk Evaluation (EuroSCORE) II, preprocedural estimated glomerular filtration rate, preprocedural hemoglobin level, prior transient ischemic attack (TIA) or stroke, peripheral artery disease and oral anticoagulation (OAC), and/or double antiplatelet therapy (DAPT) use at baseline.

Descriptive and comparative statistics were first applied to the overall study population, followed by the transfemoral TAVR cohort in whom an upper arm or femoral vein approach was used, and finally in the 1:1 PS-matched cohort. A two-tailed *p*-value < 0.05 was considered statistically significant for all tests. All analyses were performed in SPSS Statistics version 27.0.1.0 (IBM Corporation, Armonk, NY, USA) and R version 4.1.2 (R Foundation for Statistical Computing, Vienna, Austria).

## 3. Results

### 3.1. Baseline and Procedural Characteristics

Between January 2020 and January 2023, a total of 557 patients underwent TAVR. In 255 (45.8%) patients, an upper arm vein was used as a temporary pacemaker access site, in 191 (34.3%) patients the femoral vein was used, and in 111 (19.9%) patients the jugular vein was used. The baseline and procedural characteristics of the overall study population are shown in Table 1. Mean age was 78.4 ± 6.2 years, and 324 (58.2%) patients were men. Baseline characteristics were well balanced between the three cohorts, except for body mass index (BMI), coronary artery disease, peripheral artery disease, and mean EuroSCORE II, which were all significantly higher in the jugular cohort. Mean age was lowest in the jugular cohort, while the proportion of prior pacemaker implantations was lowest in the upper arm cohort.

Transfemoral TAVR was the predominant approach in the overall study population (86.2%), followed by the transaxillary (10.8%) and transapical (3.1%) approaches. The number of patients undergoing transaxillary or transapical TAVR was significantly higher in the jugular cohort compared with the upper arm and femoral cohorts. All patients in the jugular cohort underwent TAVR under general anesthesia, compared with 16.1% and 19.9% in the upper arm and femoral cohorts, respectively. Details on the significant between-group differences in the overall study population are displayed in Tables S1 and S2.

Exclusion of patients in whom the jugular vein was used, and those who underwent transaxillary or transapical TAVR, yielded an unmatched cohort of 429 transfemoral TAVR patients in whom an upper arm (*n* = 248) or femoral (*n* = 181) vein was used as the pacemaker access site. No significant differences in baseline characteristics nor between the use of general anesthesia were observed for this study population (Table 2).

**Figure 2:**
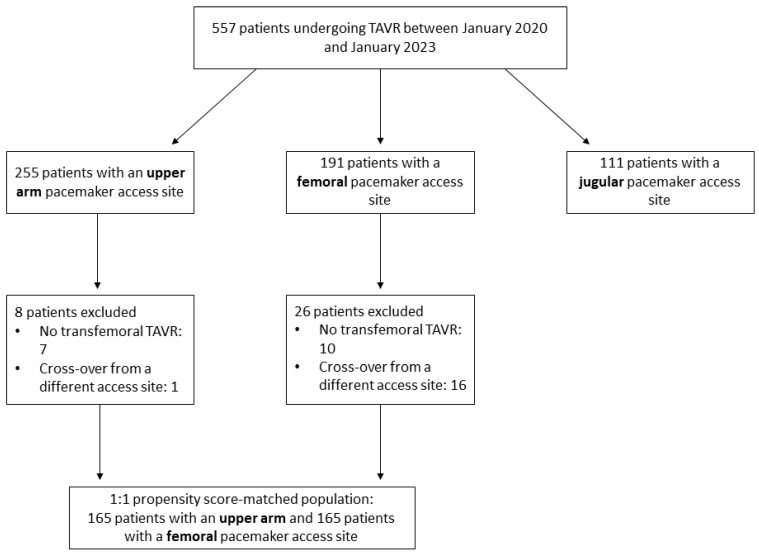
Flowchart of the study. Flowchart of the study. In the case of a cross-over of the pacemaker access site, the final access (i.e., the access site used during TAVR procedure) was scored. TAVR = transcatheter aortic valve replacement.

PS matching between upper arm and femoral vein patients resulted in 165 pairs (Figure 2). The baseline and procedural characteristics of the PS-matched cohorts were well balanced (Table 3). Matching yielded a standardized mean difference (SMD) of 0.064. Detailed information on the distribution and balance of the PS across upper arm and femoral cases is depicted in Table S3.

### 3.2. Clinical Outcomes

The clinical outcomes of the overall study population are reported in Table 1. The primary outcome of clinically relevant (i.e., BARC type 2, 3, or 5) pacemaker access site-related bleeding occurred most frequently in the jugular cohort (10.8%), followed by the femoral (5.8%) and upper arm (2.4%) cohorts (*p* = 0.003). Of note, all these bleedings were BARC type 2 bleedings, which are minor bleedings. Median time to mobilization after TAVR was significantly shortest in the upper arm cohort (480 min), followed by the femoral (1140 min) and jugular (1440 min) cohorts (*p* < 0.001). Clinically relevant bleeding not related to the pacemaker access site and the VARC-3 early safety composite outcome were comparable between the three cohorts. A cross-over of the pacemaker access site was required in 17 patients, which all occurred in the upper arm cohort (6.3% in patients in whom an upper arm approach was attempted). In 16 of these patients, a cross-over to the femoral vein was performed, and in 1 patient, a cross-over to a contralateral upper arm vein was performed. The MDRD-GFR (odds ratio [OR] 0.98, 95% confidence interval [CI]: 0.96–1.00 for every unit increase in estimated GFR; *p* = 0.02) and a non-upper-arm pacemaker access site (OR 3.50, 95% CI: 1.39–8.78; *p* = 0.008) were independently associated with the occurrence of pacemaker access site-related bleeding (Table 4).

Table 2 depicts the clinical outcomes in the unmatched transfemoral TAVR cohort. No significant differences between the upper arm and femoral cohorts were observed, except for time to mobilization (480 vs. 1140 min, *p* < 0.001).

**Figure 3:**
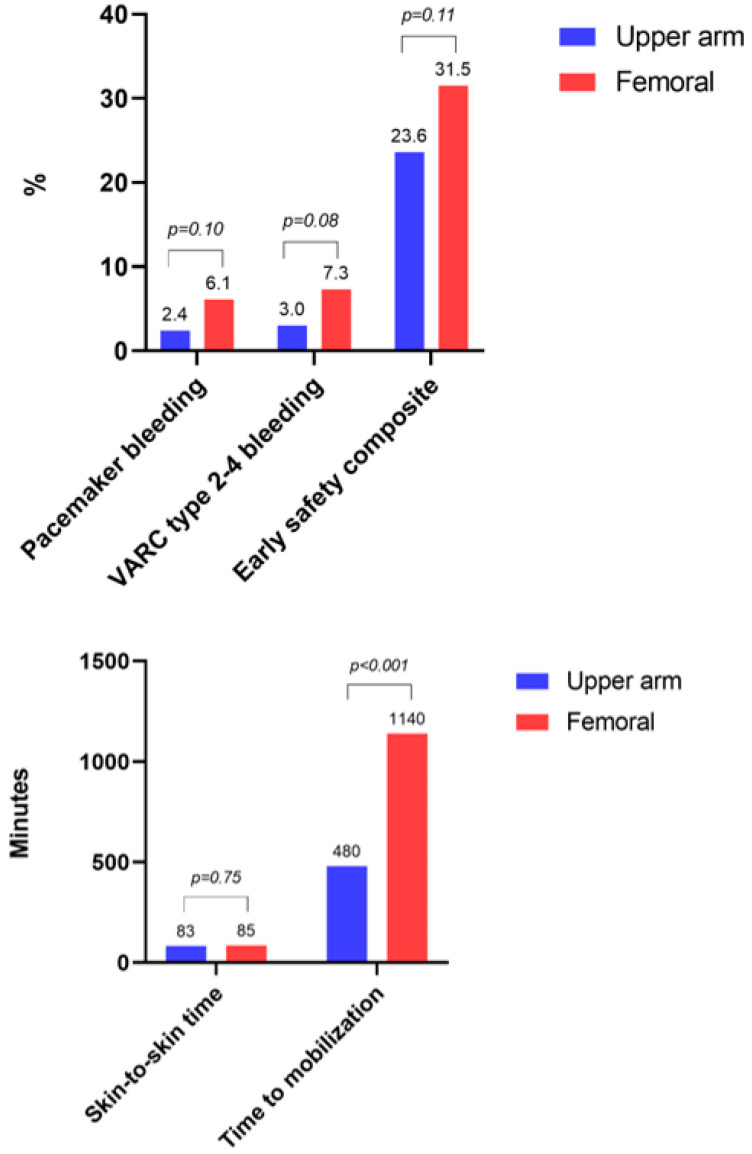
Main safety and efficacy outcomes of the study. **Upper part**: main categorical outcomes. **Lower part**: main continuous outcomes. VARC = Valve Academic Research Consortium.

Clinical outcomes in the PS-matched population are displayed in Table 3. The incidence of pacemaker access site-related bleeding was numerically lower in the upper arm cohort compared with the femoral cohort (2.4% vs. 6.1%, *p* = 0.10) (Figure 3). The median time to mobilization after TAVR was significantly shorter in the upper arm cohort compared with the femoral cohort (480 vs. 1140 min, *p* < 0.001). The mean duration of hospitalization (5.1 vs. 5.4 days, *p* = 0.50), skin-to-skin time (83 vs. 85 min, *p* = 0.75), and fluoroscopy time (19 vs. 16 min, *p* = 0.11) did not differ between both groups. The combined incidence of BARC type 2, 3, or 5 bleeding not related to the pacemaker access site was comparable between both groups (18.8% vs. 20.6%, *p* = 0.68). The VARC-3 defined early safety composite outcome was not significantly different (upper arm: 23.6% vs. femoral: 31.5%, *p* = 0.11).

## 4. Discussion

In this study, we sought to evaluate the safety and efficacy of an upper arm vein as a temporary pacemaker access site in patients undergoing TAVR. The key findings can be summarized as follows. First, in this prospective non-selective cohort of consecutive TAVR patients, the incidence of clinically relevant pacemaker access site-related bleeding was highest in the jugular cohort (10.8%), followed by the femoral (5.8%) and upper arm (2.4%) cohorts. Decreased renal function and a non-upper-arm pacemaker access site were independently associated with the primary outcome. In the PS-matched cohort, pacemaker access site-related bleeding was numerically lower in the upper arm cohort compared with the femoral cohort (2.4 vs. 6.1%). Although this difference was not statistically significant, we observed a trend toward significance. Second, time to mobilization after TAVR was significantly shorter in the upper arm cohort compared with the femoral cohort (480 vs. 1140 min). Third, the cross-over rate from an upper arm pacemaker access site was low, occurring in approximately 6% of patients.

Although procedural TAVR complications have importantly decreased over time, clinically relevant bleeding remains a frequently encountered complication with a potential detrimental impact on outcome [18]. Therefore, every attempt to decrease bleeding complications should be encouraged. With reduced sheath sizes and improved percutaneous closure devices, the bleeding complications of the primary access site have gradually diminished. On the contrary, little attention has been paid to the secondary access sites (i.e., diagnostic and pacemaker access site), and data on bleeding complications and methods to reduce secondary access site-related bleeding remain scarce. Although some studies have reported beneficial outcomes when using an alternative diagnostic access site (e.g., distal radial access) [19,20], robust evidence from randomized trials is still lacking.

To the best of our knowledge, this is the first study describing the results of an upper arm vein as an alternative temporary pacemaker access site during TAVR. Our study shows that the incidence of pacemaker access site-related bleeding of an upper arm vein is low and, if present, only minor (i.e., BARC type 2 bleeding). The observed numerical bleeding reduction of approximately 4% with the upper arm approach is in line with previously published data, which compared an upper arm approach to a femoral approach in patients undergoing right heart catheterization [8]. Furthermore, we found a shorter time to mobilization after an upper arm approach compared with a femoral approach. This difference is explained by the inability to mobilize when the femoral pacing lead is still in place, while patients with an upper arm pacing lead are allowed to mobilize due to strong fixation of the pacing lead to the upper arm. Early mobilization has been associated with a lower incidence of postoperative delirium (POD) [11]. As POD is known to adversely affect mortality in both the short and long term, rapid mobilization should be pursued. In addition, a shorter duration of immobilization may reduce the need for urinary catheterization and, hence, the incidence of urinary tract infection [10]. A shorter time to mobilization might be associated with a shorter duration of hospitalization, which in turn could diminish the burden on healthcare resources and reduce healthcare costs. In our study, the mean duration of hospitalization in the upper arm cohort was slightly shorter, although not significant. Importantly, the upper arm approach was not associated with longer skin-to-skin time.

There were 17 cases in which a cross-over of the pacemaker access site occurred. These were all cases where an initial upper arm approach was attempted, yielding an upper arm access success rate of 94%. Except for one case, all cross-overs occurred directly (i.e., at the beginning of the TAVR procedure), mostly due to a small diameter of the upper arm vein or a close proximity to the brachial artery. One late cross-over occurred. In this patient, the pacing lead dislocated approximately 1 h post-TAVR, after which a successful repositioning of the pacing lead through the femoral vein was performed in the catheterization laboratory.

Temporary pacing lead placement in an upper arm vein closely resembles the placement of a peripherally inserted central catheter (PICC) line. Although PICC lines are frequently used, these lines are known to pose an increased risk of deep vein thrombosis (DVT), with incidences between 5 and 15 percent for hospitalized patients and between 2 and 5 percent for ambulatory patients [21,22]. However, it should be noted that the incidences described in these studies concern patients in whom the PICC lines are kept significantly longer in situ (weeks) rather than the maximum of 48 h in the TAVR patients described in our cohort. In addition, patients with an indication for PICC line placement usually have a higher burden of prothrombotic risk factors (i.e., malignancy). Taking the aforementioned into account, a direct translation between incidences of DVT secondary to PICC line placement and DVT secondary to temporary pacing lead placement cannot be made. More importantly, there was no clinical suspicion of DVT in any of the patients from our study.

In attempts to further refine the TAVR procedure, some centers have adopted pacing over the left ventricular (LV) stiff wire as a primary rapid pacing strategy [23]. The drawback of such an approach is the relatively unstable contact between wire and LV wall possibly leading to the loss of pacing conduction, especially during balloon inflation when the wire position in the left ventricular outflow tract is influenced by the unfolding of the balloon. Therefore, in most cases, a back-up femoral venous sheath is placed to be able to quickly insert a temporary pacing lead if needed, introducing the same bleeding risk in these patients. In our center, we do not use pacing over the wire as a standard rapid pacing strategy.

Antithrombotic agents are frequently prescribed in patients undergoing TAVR. Due to the high prevalence of coronary artery disease and atrial fibrillation, OAC and DAPT use is particular prevalent in the elderly TAVR population [24]. Antithrombotic agents in general, but especially OAC and/or DAPT use, are known to increase the risk of periprocedural bleeding complications [16,17]. Although the number of patients with OAC and/or DAPT use at baseline was slightly lower in the upper arm cohort compared with the femoral cohort, after matching on this covariate, pacemaker access site-related bleeding still occurred numerically less in the upper arm cohort.

### 4.1. Future Perspectives

With the annually increasing number of TAVR procedures worldwide, there is an ongoing need to further reduce the invasiveness and complications of TAVR procedures [25]. As bleeding complications are still frequently encountered, future studies aiming to reduce periprocedural bleeding complications are warranted. We initiated the multicenter randomized TAVI XS trial (ClinicalTrials.gov: NCT05672823) which investigates whether an upper extremity approach, using the radial artery for diagnostic access and an upper arm vein for temporary pacemaker access, reduces bleeding complications compared with a lower extremity approach using the femoral artery and femoral vein for diagnostic and pacemaker access, respectively. With the adoption of the recommendation to omit clopidogrel after TAVR in patients with and without an indication for chronic anticoagulant therapy (both in American and European guidelines) [26,27], a further decrease in periprocedural bleeding complications might be expected.

### 4.2. Study Limitations

This study has some limitations. First, due to the observational nature of this study, the results should be interpreted with caution as no randomized comparison was made between the upper arm and femoral approach. We performed PS matching to overcome differences in baseline characteristics and potential confounders. However, a residual impact of unknown or unmeasured confounding factors cannot be excluded. Second, we did not routinely perform an ultrasound of the extremities to assess the incidence of DVT. Although there were no cases with a clinical suspicion of DVT, we cannot exclude cases with subclinical DVT. Third, no PS-matched analysis was performed between the upper arm and jugular cohort. However, to provide some insights into the latter category, baseline characteristics, procedural characteristics, and clinical outcomes are provided for this patient cohort as well.

## 5. Conclusions

In conclusion, we have demonstrated that the use of an upper arm vein as a temporary pacemaker access site is safe and feasible, and that its use might be associated with fewer bleeding complications and a shorter time to mobilization compared with the femoral vein.

## Figures and Tables

**Table 1:** Baseline characteristics, procedural characteristics, and clinical outcomes in the overall study population.

	Total Cohort (*n* = 557)	Upper Arm Cohort (*n* = 255)	Femoral Cohort (*n* = 191)	Jugular Cohort (*n* = 111)	*p* Value
**Baseline characteristics**					
Age, years	78.4 ± 6.2	78.7 ± 5.9	78.7 ± 6.2	77.0 ± 6.9	**0.03**
Male sex, *n* (%)	324 (58.2)	151 (59.2)	104 (54.5)	69 (62.2)	0.38
BMI, kg/m^2^	27.5 ± 5.0	27.5 ± 4.7	26.9 ± 4.7	28.4 ± 6.0	**0.04**
NYHA class III/IV, *n* (%)	148 (26.6)	67 (26.3)	47 (24.6)	34 (30.6)	0.52
EuroSCORE II	2.92 ± 2.54	2.54 ± 1.89	3.07 ± 2.84	3.52 ± 3.13	**0.002**
Coronary artery disease, *n* (%)	287 (51.5)	120 (47.1)	98 (51.3)	69 (62.2)	**0.03**
Peripheral artery disease, *n* (%)	107 (19.2)	31 (12.2)	30 (15.7)	46 (41.4)	**<0.001**
Previous stroke or TIA, *n* (%)	97 (17.4)	49 (19.2)	30 (15.7)	18 (16.2)	0.59
Prior pacemaker implantation, *n* (%)	39 (7.0)	8 (3.1)	24 (12.6)	7 (6.3)	**<0.001**
LBBB, *n* (%)	65 (11.7)	33 (12.9)	26 (13.6)	6 (5.4)	0.07
RBBB, *n* (%)	51 (9.2)	26 (10.2)	15 (7.9)	10 (9.0)	0.67
MDRD-GFR, mL/min	63 ± 19	63 ± 19	62 ± 20	63 ± 19	0.64
Hemoglobin level, mmol/L	7.9 ± 1.0	8.0 ± 1.0	7.9 ± 0.9	7.9 ± 0.9	0.28
OAC and/or DAPT use at baseline, *n* (%)	304 (54.6)	133 (52.2)	107 (56.0)	64 (57.7)	0.55
**Procedural characteristics**					
TAVR approach					
- Transfemoral, *n* (%)	480 (86.2)	248 (97.3)	181 (94.8)	51 (45.9)	**<0.001**
- Transaxillary, *n* (%)	60 (10.8)	7 (2.7)	10 (5.2)	43 (38.7)	**<0.001**
- Transapical, *n* (%)	17 (3.1)	0	0	17 (15.3)	**<0.001**
Valve type					
- Self-expanding, *n* (%)	471 (84.6)	214 (83.9)	167 (87.4)	90 (81.1)	0.31
- Balloon-expandable, *n* (%)	86 (15.4)	41 (16.1)	24 (12.6)	21 (18.9)	0.31
General anesthesia, *n* (%)	190 (34.1)	41 (16.1)	38 (19.9)	111 (100)	**<0.001**
**Primary outcome**					
BARC type 2, 3, or 5 pacemaker access site-related bleeding, *n* (%)	29 (5.2)	6 (2.4)	11 (5.8)	12 (10.8)	**0.003**
**Secondary efficacy outcomes**					
Time to mobilization after TAVR, min	720 [360–1440]	480 [360–1440]	1140 [487.5–1440]	1440 [690–1830]	**<0.001**
Duration of hospitalization, days	5.5 ± 4.1	4.9 ± 3.8	5.6 ± 4.1	6.5 ± 4.7	**0.003**
Skin-to-skin time, min	87 ± 41	85 ± 39	86 ± 41	91 ± 44	0.49
Fluoroscopy time, min	17 ± 11	18 ± 13	17 ± 9	15 ± 8	0.07
Duration pacing lead in place, min	1031 ± 1325	993 ± 883	756 ± 1222	1601 ± 2028	**<0.001**
Dysfunction pacemaker, *n* (%)	35 (6.3)	17 (6.7)	11 (5.8)	7 (6.3)	0.92
Back-up pacing required, *n* (%)	85 (15.3)	42 (16.5)	26 (13.6)	17 (15.3)	0.70
Cross-over pacemaker access site	17	17	0	0	N/A
**Secondary safety outcomes**					
BARC type 2, 3, or 5 bleeding not related to pacemaker access site, *n* (%)	104 (18.7)	48 (18.8)	38 (19.9)	18 (16.2)	0.73
VARC-3 early safety composite, *n* (%)	162 (29.1)	64 (25.1)	63 (33.0)	35 (31.5)	0.16

Data are presented as mean ± standard deviation, median [interquartile range], or as a number (%). BARC = Bleeding Academic Research Consortium; BMI = body mass index; DAPT = double antiplatelet therapy; EuroSCORE = European System for Cardiac Operative Risk Evaluation; MDRD-GFR = Modification of Diet in Renal Disease–glomerular filtration rate; LBBB = left bundle branch block; NYHA = New York Heart Association; OAC = oral anticoagulation; RBBB = right bundle branch block; TAVR = transcatheter aortic valve replacement; TIA = transient ischemic attack; VARC = Valve Academic Research Consortium.

**Table 2:** Baseline characteristics, procedural characteristics, and clinical outcomes in the unmatched population of patients undergoing transfemoral TAVR using an upper arm or femoral vein approach.

	Total Cohort (*n* = 429)	Upper Arm Cohort (*n* = 248)	Femoral Cohort (*n* = 181)	*p* Value
**Baseline and procedural characteristics**				
Age, years	79.0 ± 5.8	78.9 ± 5.8	79.2 ± 5.8	0.69
Male sex, *n* (%)	242 (56.4)	144 (58.1)	98 (54.1)	0.42
BMI, kg/m^2^	27.2 ± 4.7	27.5 ± 4.7	26.8 ± 4.8	0.15
NYHA class III/IV, *n* (%)	106 (24.7)	65 (26.2)	41 (22.7)	0.40
EuroSCORE II	2.70 ± 2.13	2.54 ± 1.91	2.91 ± 2.40	0.08
Coronary artery disease, *n* (%)	204 (47.6)	114 (46.0)	90 (49.7)	0.44
Peripheral artery disease, *n* (%)	48 (11.2)	25 (10.1)	23 (12.7)	0.39
Previous stroke or TIA, *n* (%)	74 (17.2)	48 (19.4)	26 (14.4)	0.18
Prior pacemaker implantation, *n* (%)	32 (7.5)	8 (3.2)	24 (13.3)	**<0.001**
LBBB, *n* (%)	55 (12.8)	30 (12.1)	25 (13.8)	0.57
RBBB, *n* (%)	40 (9.3)	25 (10.1)	15 (8.3)	0.57
MDRD-GFR, mL/min	63 ± 19	63 ± 19	62 ± 19	0.43
Hemoglobin level, mmol/L	7.9 ± 1.0	8.0 ± 1.0	7.9 ± 0.9	0.34
OAC and/or DAPT use at baseline, *n* (%)	229 (53.4)	128 (51.6)	101 (55.8)	0.39
Valve type				
- Self-expanding, *n* (%)	365 (85.1)	208 (83.9)	157 (86.7)	0.41
- Balloon-expandable, *n* (%)	64 (14.9)	40 (16.1)	24 (13.3)	0.41
General anesthesia, *n* (%)	62 (14.5)	34 (13.7)	28 (15.5)	0.61
**Primary outcome**				
BARC type 2, 3, or 5 pacemaker access site-related bleeding, *n* (%)	17 (4.0)	6 (2.4)	11 (6.1)	0.06
**Secondary efficacy outcomes**				
Time to mobilization after TAVR, min	600 [360–1440]	480 [360–1440]	1140 [431.25–1440]	**<0.001**
Duration of hospitalization, days	5.1 ± 3.9	4.9 ± 3.9	5.3 ± 3.9	0.29
Skin-to-skin time, min	85 ± 40	84 ± 38	85 ± 41	0.83
Fluoroscopy time, min	18 ± 12	18 ± 13	17 ± 9	0.33
Duration pacing lead in place, min	872 ± 1053	976 ± 885	726 ± 1238	**0.02**
Dysfunction pacemaker, *n* (%)	28 (6.5)	17 (6.9)	11 (6.1)	0.75
Back-up pacing required, *n* (%)	67 (15.6)	41 (16.5)	26 (14.4)	0.53
Cross-over pacemaker access site, *n*	17	17	0	N/A
**Secondary safety outcomes**				
BARC type 2, 3, or 5 bleeding not related to pacemaker access site, *n* (%)	85 (19.8)	47 (19.0)	38 (21.0)	0.60
VARC-3 early safety composite, *n* (%)	121 (28.2)	63 (25.4)	58 (32.0)	0.13

Data are presented as mean ± standard deviation, median [interquartile range], or as a number (%). BARC = Bleeding Academic Research Consortium; BMI = body mass index; DAPT = double antiplatelet therapy; EuroSCORE = European System for Cardiac Operative Risk Evaluation; MDRD-GFR = Modification of Diet in Renal Disease–glomerular filtration rate; LBBB = left bundle branch block; NYHA = New York Heart Association; OAC = oral anticoagulation; RBBB = right bundle branch block; TAVR = transcatheter aortic valve replacement; TIA = transient ischemic attack; VARC = Valve Academic Research Consortium.

**Table 3:** Baseline characteristics, procedural characteristics, and clinical outcomes in the propensity score-matched population of patients undergoing transfemoral TAVR using an upper arm or femoral vein approach.

	Upper Arm Cohort (*n* = 165)	Femoral Cohort (*n* = 165)	*p* Value
**Baseline and procedural characteristics**			
Age, years	79.3 ± 5.6	79.0 ± 5.7	0.69
Male gender, *n* (%)	87 (52.7)	93 (56.4)	0.51
BMI, kg/m^2^	27.5 ± 4.4	26.9 ± 4.8	0.25
NYHA class III/IV, *n* (%)	49 (29.7)	38 (23.0)	0.17
EuroSCORE II	2.87 ± 2.18	2.97 ± 2.47	0.70
Coronary artery disease, *n* (%)	80 (48.5)	83 (50.3)	0.74
Peripheral artery disease, *n* (%)	24 (14.5)	23 (13.9)	0.88
Previous stroke or TIA, *n* (%)	29 (17.6)	26 (15.8)	0.66
Prior pacemaker implantation, *n* (%)	6 (3.6)	24 (14.5)	**<0.001**
LBBB, *n* (%)	16 (9.7)	24 (14.5)	0.19
RBBB, *n* (%)	15 (9.1)	13 (7.9)	0.77
MDRD-GFR, mL/min	62 ± 19	62 ± 19	0.84
Hemoglobin level, mmol/L	8.0 ± 1.0	7.9 ± 0.9	0.58
OAC and/or DAPT use at baseline, *n* (%)	87 (52.7)	92 (55.8)	0.58
Valve type			
- Self-expanding, *n* (%)	142 (86.1)	145 (87.9)	0.62
- Balloon-expandable, *n* (%)	23 (13.9)	20 (12.1)	0.62
General anesthesia, *n* (%)	21 (12.7)	27 (16.4)	0.35
**Primary outcome**			
BARC type 2, 3, or 5 pacemaker access site-related bleeding, *n* (%)	4 (2.4)	10 (6.1)	0.10
**Secondary efficacy outcomes**			
Time to mobilization after TAVR, min	480 [360–1440]	1140 [420–1440]	**<0.001**
Duration of hospitalization, days	5.1 ± 4.4	5.4 ± 3.9	0.50
Skin-to-skin time, min	83 ± 36	85 ± 41	0.75
Fluoroscopy time, min	19 ± 15	16 ± 9	0.11
Duration pacing lead in place, min	1002 ± 948	745 ± 1276	**0.04**
Dysfunction pacemaker, *n* (%)	12 (7.3)	9 (5.5)	0.49
Back-up pacing required, *n* (%)	26 (15.8)	24 (14.5)	0.76
**Secondary safety outcomes**			
BARC type 2, 3, or 5 not related to pacemaker access site, *n* (%)	31 (18.8)	34 (20.6)	0.68
- BARC type 2, *n* (%)	26 (15.8)	21 (12.7)	0.43
- BARC type 3, *n* (%)	5 (3.0)	11 (6.7)	0.12
- BARC type 5, *n* (%)	0	2 (1.2)	0.50
Early safety outcomes (at 30 days)			
- All-cause mortality, *n* (%)	2 (1.2)	5 (3.0)	0.45
- All stroke, *n* (%)	7 (4.2)	12 (7.3)	0.24
- VARC type 2–4 bleeding, *n* (%)	5 (3.0)	12 (7.3)	0.08
- Major vascular, access-related, or cardiac structural complication, *n* (%)	4 (2.4)	9 (5.5)	0.16
- Moderate or severe aortic regurgitation, *n* (%)	5 (3.0)	10 (6.1)	0.19
- New permanent pacemaker implantation, *n* (%)	20 (12.1)	21 (12.7)	0.87
- Surgery or intervention related to device, *n* (%)	0	0	0
- Early safety composite, *n* (%)	39 (23.6)	52 (31.5)	0.11

Data are presented as mean ± standard deviation, median [interquartile range], or as a number (%). BARC = Bleeding Academic Research Consortium; BMI = body mass index; DAPT = double antiplatelet therapy; EuroSCORE = European System for Cardiac Operative Risk Evaluation; MDRD-GFR = Modification of Diet in Renal Disease–glomerular filtration rate; LBBB = left bundle branch block; NYHA = New York Heart Association; OAC = oral anticoagulation; RBBB = right bundle branch block; TAVR = transcatheter aortic valve replacement; TIA = transient ischemic attack.

**Table 4:** Baseline characteristics associated with pacemaker access site-related bleeding in the overall study population.

	Univariate Analyses OR (95% CI)	*p* Value	Multivariate Analyses OR (95% CI)	*p* Value
Age	1.05 (0.98–1.12)	**0.17**	1.05 (0.99–1.12)	0.12
Female sex	1.14 (0.54–2.41)	0.74		
NYHA class III/IV	1.06 (0.46–2.44)	0.90		
EuroSCORE II	1.00 (0.87–1.16)	0.99		
Coronary artery disease	1.01 (0.48–2.13)	0.98		
Peripheral artery disease	1.10 (0.44–2.78)	0.84		
Prior stroke/TIA	1.25 (0.50–3.16)	0.63		
MDRD-GFR, mL/min	0.98 (0.96–1.00)	**0.02**	0.98 (0.96–1.00)	**0.02**
Hemoglobin level, mmol/L	0.84 (0.57–1.24)	0.37		
OAC and/or DAPT use at baseline	1.03 (0.48–2.18)	0.95		
Pacemaker access site				
- Upper arm	Reference		Reference	
- Femoral	2.54 (0.92–6.99)	0.07	2.47 (0.89–6.85)	0.08
- Jugular	5.03 (1.84–13.78)	**0.002**	5.68 (2.04–15.80)	**<0.001**
Pacemaker access site				
- Upper arm	Reference		Reference	
- Non-upper-arm	3.42 (1.37–8.54)	**0.008**	3.50 (1.39–8.78)	**0.008**

DAPT = double antiplatelet therapy; EuroSCORE = European System for Cardiac Operative Risk Evaluation; MDRD-GFR = Modification of Diet in Renal Disease–glomerular filtration rate; NYHA = New York Heart Association; OAC = oral anticoagulation; OR = odds ratio; TIA = transient ischemic attack.

## Data Availability

The data presented in this study are available on request from the corresponding author.

## Supplementary Materials

The following supporting information can be downloaded at: https://www.mdpi.com/article/10.3390/jcm13030651/s1, Figure S1: Distribution of the three different pacemaker access sites over time; Table S1: Post-hoc testing of variables in the overall study population with significant findings after ANOVA testing; Table S2: Post-hoc testing of variables in the overall study population with significant findings after Chi-Square testing; Table S3: Standardized mean difference for baseline characteristics used for propensity score matching before and after matching.

## Abbreviations

AS	aortic stenosis
BARC	Bleeding Academic Research Consortium
POD	postoperative delirium
PS	propensity score
TAVR	transcatheter aortic valve replacement
VARC	Valve Academic Research Consortium

## References

[1] Smith C.R., Leon M.B., Mack M.J., Miller D.C., Moses J.W., Svensson L.G., Tuzcu E.M., Webb J.G., Fontana G.P., Makkar R.R. (2011). Transcatheter versus surgical aortic-valve replacement in high-risk patients. N. Engl. J. Med..

[2] Adams D.H., Popma J.J., Reardon M.J., Yakubov S.J., Coselli J.S., Deeb G.M., Gleason T.G., Buchbinder M., Hermiller J., Kleiman N.S. (2014). Transcatheter aortic-valve replacement with a self-expanding prosthesis. N. Engl. J. Med..

[3] Leon M.B., Smith C.R., Mack M.J., Makkar R.R., Svensson L.G., Kodali S.K., Thourani V.H., Tuzcu E.M., Miller D.C., Herrmann H.C. (2016). Transcatheter or Surgical Aortic-Valve Replacement in Intermediate-Risk Patients. N. Engl. J. Med..

[4] Reardon M.J., Van Mieghem N.M., Popma J.J., Kleiman N.S., Søndergaard L., Mumtaz M., Adams D.H., Deeb G.M., Maini B., Gada H. (2017). Surgical or Transcatheter Aortic-Valve Replacement in Intermediate-Risk Patients. N. Engl. J. Med..

[5] Mack M.J., Leon M.B., Thourani V.H., Makkar R., Kodali S.K., Russo M., Kapadia S.R., Malaisrie S.C., Cohen D.J., Pibarot P. (2019). Transcatheter Aortic-Valve Replacement with a Balloon-Expandable Valve in Low-Risk Patients. N. Engl. J. Med..

[6] Popma J.J., Deeb G.M., Yakubov S.J., Mumtaz M., Gada H., O’Hair D., Bajwa T., Heiser J.C., Merhi W., Kleiman N.S. (2019). Transcatheter Aortic-Valve Replacement with a Self-Expanding Valve in Low-Risk Patients. N. Engl. J. Med..

[7] Rodés-Cabau J., Ellenbogen K.A., Krahn A.D., Latib A., Mack M., Mittal S., Muntané-Carol G., Nazif T.M., Sondergaard L., Urena M. (2019). Management of Conduction Disturbances Associated with Transcatheter Aortic Valve Replacement: JACC Scientific Expert Panel. J. Am. Coll. Cardiol..

[8] Roule V., Ailem S., Legallois D., Dahdouh Z., Lognoné T., Bergot E., Grollier G., Milliez P., Sabatier R., Beygui F. (2015). Antecubital vs. Femoral Venous Access for Right Heart Catheterization: Benefits of a Flashback. Can. J. Cardiol..

[9] Lim T., Ryu H.G., Jung C.W., Jeon Y., Bahk J.H. (2012). Effect of the bevel direction of puncture needle on success rate and complications during internal jugular vein catheterization. Crit. Care Med..

[10] Lauck S.B., Kwon J.Y., Wood D.A., Baumbusch J., Norekvål T.M., Htun N., Stephenson L., Webb J.G. (2018). Avoidance of urinary catheterization to minimize in-hospital complications after transcatheter aortic valve implantation: An observational study. Eur. J. Cardiovasc. Nurs..

[11] van der Wulp K., van Wely M.H., Rooijakkers M.J.P., Brouwer M.A., van den Boogaard M., Pickkers P., Olde Rikkert M.G.M., Delewi R., Van Mieghem N.M., Baan J. (2020). Delirium After TAVR: Crosspassing the Limit of Resilience. JACC Cardiovasc. Interv..

[12] Généreux P., Piazza N., Alu M.C., Nazif T., Hahn R.T., Pibarot P., Bax J.J., Leipsic J.A., Blanke P., Blackstone E.H. (2021). Valve Academic Research Consortium 3: Updated endpoint definitions for aortic valve clinical research. Eur. Heart J..

[13] Ho D., Imai K., King G., Stuart E.A. (2011). MatchIt: Nonparametric Preprocessing for Parametric Causal Inference. J. Stat. Softw..

[14] Hansen B.B., Klopfer S.O. (2006). Optimal Full Matching and Related Designs via Network Flows. J. Comput. Graph. Stat..

[15] Sherwood M.W., Xiang K., Matsouaka R., Li Z., Vemulapalli S., Vora A.N., Fanaroff A., Harrison J.K., Thourani V.H., Holmes D. (2020). Incidence, Temporal Trends, and Associated Outcomes of Vascular and Bleeding Complications in Patients Undergoing Transfemoral Transcatheter Aortic Valve Replacement: Insights From the Society of Thoracic Surgeons/American College of Cardiology Transcatheter Valve Therapies Registry. Circ. Cardiovasc. Interv..

[16] Navarese E.P., Zhang Z., Kubica J., Andreotti F., Farinaccio A., Bartorelli A.L., Bedogni F., Rupji M., Tomai F., Giordano A. (2021). Development and Validation of a Practical Model to Identify Patients at Risk of Bleeding After TAVR. JACC Cardiovasc. Interv..

[17] Mangieri A., Montalto C., Poletti E., Sticchi A., Crimi G., Giannini F., Latib A., Capodanno D., Colombo A. (2019). Thrombotic Versus Bleeding Risk After Transcatheter Aortic Valve Replacement: JACC Review Topic of the Week. J. Am. Coll. Cardiol..

[18] Avvedimento M., Nuche J., Farjat-Pasos J.I., Rodés-Cabau J. (2023). Bleeding Events After Transcatheter Aortic Valve Replacement: JACC State-of-the-Art Review. J. Am. Coll. Cardiol..

[19] Achim A., Szűcsborus T., Sasi V., Nagy F., Jambrik Z., Nemes A., Varga A., Bertrand O.F., Ruzsa Z. (2022). Distal Radial Secondary Access for Transcatheter Aortic Valve Implantation: The Minimalistic Approach. Cardiovasc. Revasc. Med..

[20] Achim A., Szigethy T., Olajos D., Molnár L., Papp R., Bárczi G., Kákonyi K., Édes I.F., Becker D., Merkely B. (2022). Switching From Proximal to Distal Radial Artery Access for Coronary Chronic Total Occlusion Recanalization. Front. Cardiovasc. Med..

[21] Taxbro K., Hammarskjöld F., Thelin B., Lewin F., Hagman H., Hanberger H., Berg S. (2019). Clinical impact of peripherally inserted central catheters vs implanted port catheters in patients with cancer: An open-label, randomised, two-centre trial. Br. J. Anaesth..

[22] Fallouh N., McGuirk H.M., Flanders S.A., Chopra V. (2015). Peripherally Inserted Central Catheter-associated Deep Vein Thrombosis: A Narrative Review. Am. J. Med..

[23] Blusztein D., Raney A., Walsh J., Nazif T., Woods C., Daniels D. (2023). Best Practices in Left Ventricular Pacing for Transcatheter Aortic Valve Replacement. Struct. Heart.

[24] Capodanno D., Collet J.P., Dangas G., Montalescot G., Ten Berg J.M., Windecker S., Angiolillo D.J. (2021). Antithrombotic Therapy After Transcatheter Aortic Valve Replacement. JACC Cardiovasc. Interv..

[25] Carroll J.D., Mack M.J., Vemulapalli S., Herrmann H.C., Gleason T.G., Hanzel G., Deeb G.M., Thourani V.H., Cohen D.J., Desai N. (2020). STS-ACC TVT Registry of Transcatheter Aortic Valve Replacement. J. Am. Coll. Cardiol..

[26] Otto C.M., Nishimura R.A., Bonow R.O., Carabello B.A., Erwin J.P., Gentile F., Jneid H., Krieger E.V., Mack M., McLeod C. (2021). 2020 ACC/AHA Guideline for the Management of Patients with Valvular Heart Disease: Executive Summary: A Report of the American College of Cardiology/American Heart Association Joint Committee on Clinical Practice Guidelines. J. Am. Coll. Cardiol..

[27] Vahanian A., Beyersdorf F., Praz F., Milojevic M., Baldus S., Bauersachs J., Capodanno D., Conradi L., De Bonis M., De Paulis R. (2022). 2021 ESC/EACTS Guidelines for the management of valvular heart disease. Eur. Heart J..

