# Supplementary material for: Using Upper Arm Vein as Temporary Pacemaker Access Site: A Next Step in Minimizing the Invasiveness of Transcatheter Aortic Valve Replacement
Source: J Clin Med. 2024 Jan 23;13(3):651. doi: 10.3390/jcm13030651 (PMC10855945; doi:10.3390/jcm13030651)
Supplement: Supplementary file 1 [file jcm-13-00651-s001.zip › jcm-2738405-supplementary.pdf]

Journal of
Clinical Medicine

Article

Using Upper Arm Vein as Temporary Pacemaker Access Site: A Next Step in Minimizing the Invasiveness of Transcatheter Aortic Valve Replacement

Maxim J. P. Rooijakkers [1], Geert A. A. Versteeg [1], Marleen H. van Wely [1], Laura Rodwell [2], Lokien X. van Nunen [1], Robert Jan van Geuns [1], Leen A. F. M. van Garsse [3], Guillaume S. C. Geuzebroek [3], Michel W. A. Verkroost [3], Robin H. Heijmen [3] and Niels van Royen [1,*]

Supplementary Materials:

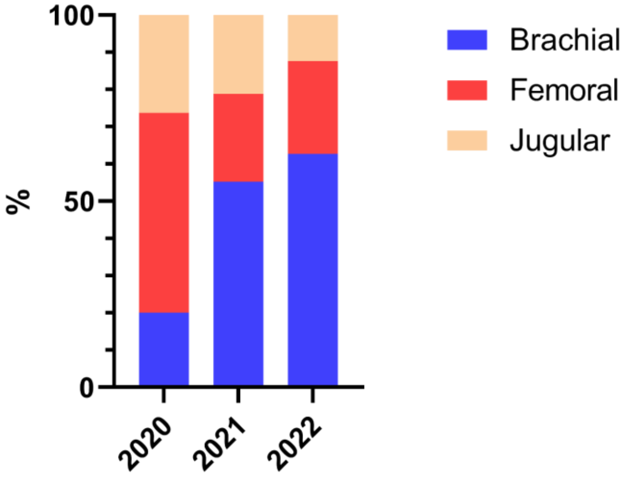

Figure S1. Distribution of the three different pacemaker access sites over time.

Table S1. Post-hoc testing of variables in the overall study population with significant findings after ANOVA testing.

	**P value ANOVA**	**Comparison between groups**	**P value post-hoc test**
Age, years	0.03	Brachial vs femoral	1.00
		Brachial vs jugular	**0.04**
		Femoral vs jugular	0.06
EuroSCORE II	0.002	Brachial vs femoral	0.08
		Brachial vs jugular	**0.002**
		Femoral vs jugular	0.41
Time to mobilization after TAVR, min	<0.001	Brachial vs femoral	**0.002**
		Brachial vs jugular	**<0.001**
		Femoral vs jugular	**0.003**
Total duration of hospitalization, days	0.003	Brachial vs femoral	0.27
		Brachial vs jugular	**0.002**
		Femoral vs jugular	0.19

ANOVA = Analysis of Variance; EuroSCORE = European System for Cardiac Operative Risk Evaluation; TAVR = transcatheter aortic valve replacement.

Table S2. Post-hoc testing of variables in the overall study population with significant findings after Chi-Square testing.

	P value Chi-Square	Comparison between groups	P value post-hoc test
Coronary artery disease	0.03	Brachial vs femoral	0.37
		Brachial vs jugular	**0.008**
		Femoral vs jugular	0.07
Peripheral artery disease	<0.001	Brachial vs femoral	0.28
		Brachial vs jugular	**<0.001**
		Femoral vs jugular	**<0.001**
Transfemoral TAVR	<0.001	Brachial vs femoral	0.17
		Brachial vs jugular	**<0.001**
		Femoral vs jugular	**<0.001**
Transaxillary TAVR	<0.001	Brachial vs femoral	0.17
		Brachial vs jugular	**<0.001**
		Femoral vs jugular	**<0.001**
Transapical TAVR	<0.001	Brachial vs femoral	N/A
		Brachial vs jugular	**<0.001**
		Femoral vs jugular	**<0.001**
General anesthesia	<0.001	Brachial vs femoral	0.30
		Brachial vs jugular	**<0.001**
		Femoral vs jugular	**<0.001**
BARC type 2, 3 or 5 pacemaker access site-related bleeding	0.003	Brachial vs femoral	0.06
		Brachial vs jugular	**<0.001**
		Femoral vs jugular	0.11

BARC = Bleeding Academic Research Consortium; TAVR = transcatheter aortic valve replacement..

Table S3. Standardized mean difference for baseline characteristics used for propensity score matching before and after matching.

	Before matching SMD	After matching SMD
Distance	0.280	0.064
Age, years	0.021	0.043
Male gender	0.039	0.074
EuroSCORE II	0.222	0.051
Peripheral artery disease	0.127	0.020
Prior stroke or TIA	0.093	0.046
MDRD-GFR, ml/min	0.082	0.024
Hemoglobin level, mmol/l	0.046	0.057
OAC and/or DAPT use at baseline	0.087	0.061

DAPT = double antiplatelet therapy; EuroSCORE = European System for Cardiac Operative Risk Evaluation; MDRD-GFR = Modification of Diet in Renal Disease – glomerular filtration rate; OAC = oral anticoagulation; SMD = standardized mean difference; TIA = transient ischemic attack.
